# Presence and activity of nitrogen-fixing bacteria in Scots pine needles in a boreal forest: a nitrogen-addition experiment

**DOI:** 10.1093/treephys/tpad048

**Published:** 2023-04-18

**Authors:** Tinkara Bizjak, Anita Sellstedt, Regina Gratz, Annika Nordin

**Affiliations:** Umeå Plant Science Centre (UPSC), Department of Forest Genetics and Plant Physiology, Swedish University of Agricultural Sciences, 901 83 Umeå, Sweden; Umeå Plant Science Centre (UPSC), Department of Plant Physiology, Umeå University, 901 87 Umeå, Sweden; Umeå Plant Science Centre (UPSC), Department of Forest Genetics and Plant Physiology, Swedish University of Agricultural Sciences, 901 83 Umeå, Sweden; Department of Forest Ecology and Management, Swedish University of Agricultural Sciences, 901 83 Umeå, Sweden; Umeå Plant Science Centre (UPSC), Department of Forest Genetics and Plant Physiology, Swedish University of Agricultural Sciences, 901 83 Umeå, Sweden

**Keywords:** diazotrophic bacteria, forest fertilization, needle endophyte, NifH, nitrogenase activity, *Pinus sylvestris*

## Abstract

Endophytic nitrogen-fixing bacteria have been detected and isolated from the needles of conifer trees growing in North American boreal forests. Because boreal forests are nutrient-limited, these bacteria could provide an important source of nitrogen for tree species. This study aimed to determine their presence and activity in a Scandinavian boreal forest, using immunodetection of nitrogenase enzyme subunits and acetylene-reduction assays of native Scots pine (*Pinus sylvestris* L.) needles. The presence and rate of nitrogen fixation by endophytic bacteria were compared between control plots and fertilized plots in a nitrogen-addition experiment. In contrast to the expectation that nitrogen-fixation rates would decline in fertilized plots, as seen, for instance, with nitrogen-fixing bacteria associated with bryophytes, there was no difference in the presence or activity of nitrogen-fixing bacteria between the two treatments. The extrapolated calculated rate of nitrogen fixation relevant for the forest stand was 20 g N ha^−1^ year^−1^, which is rather low compared with Scots pine annual nitrogen use but could be important for the nitrogen-poor forest in the long term. In addition, of 13 colonies of potential nitrogen-fixing bacteria isolated from the needles on nitrogen-free media, 10 showed in vitro nitrogen fixation. In summary, 16S rRNA sequencing identified the species as belonging to the genera *Bacillus*, *Variovorax*, *Novosphingobium*, *Sphingomonas*, *Microbacterium* and *Priestia*, which was confirmed by Illumina whole-genome sequencing. Our results confirm the presence of endophytic nitrogen-fixing bacteria in Scots pine needles and suggest that they could be important for the long-term nitrogen budget of the Scandinavian boreal forest.

## Introduction

To date, all plant species studied appear to be inhabited by endophytic bacteria (Santoyo et al. 2016), which are defined as microorganisms that reside in plants but do not cause any symptoms of disease (Wilson 1995). Endophytes can be symbionts, latent pathogens or harmless cohabitants (Chanway et al. 2014). Endophytic bacteria possess a diverse set of plant growth-promoting properties that can positively affect, for example, phytohormone balance (Bhattacharjee et al. 2008), nutrient acquisition (Santoyo et al. 2016), growth and yield (Ryan et al. 2008, Khan et al. 2012), protection against pathogens (Wei et al. 2014), propagation (Quambusch et al. 2014) and the response to abiotic stress. An important endophytic bacterial community that can help with nutrient acquisition comprises diazotrophic bacteria, which are capable of fixing nitrogen from the atmosphere. The multi-subunit enzyme responsible for the energy-demanding process of nitrogen fixation is nitrogenase (Doty et al. 2016), encoded by the structural genes *nifH*, *nifD* and *nifK*, and regulated by *nifA* (Marchal and Vanderleyden 2000). Of these genes, *nifH* is most commonly used as a marker for nitrogen-fixing bacteria (Zhang et al. 2007). Nitrogenase is extremely oxygen-sensitive (Marchal and Vanderleyden 2000) and its activity is upregulated by phosphorus (Reed et al. 2007, Matson et al. 2014), and probably also by carbohydrates because of the high energy demand of nitrogen fixation (Welsh 2000, Zheng et al. 2017, Benavides et al. 2020). It is less clear, however, how a supply of externally added mineral nitrogen fertilizer affects nitrogen fixation. Many studies have shown adverse effects of external nitrogen application or higher natural nitrogen soil availability on nitrogen fixation (Barron et al. 2011, Gundale et al. 2011, Zheng et al. 2017, Nishida and Suzaki 2018), but some have shown no effects (Moyes et al. 2016, Zheng et al. 2017). Nitrogen fixation could also be downregulated by higher nitrogen concentrations in the environment, as indicated by studies where higher nitrogen-fixation rates have been measured in tissues with a higher carbon-to-nitrogen (C/N) ratio (Granhall and Lindberg 1978, Leppänen et al. 2013, Tormanen and Smolander 2022).

In the boreal forest, where plant growth is strongly nitrogen-limited, it has been suggested that in addition to the nitrogen-fixing symbiosis between cyanobacteria and mosses (DeLuca et al. 2002), endophytic nitrogen fixation provides an important additional source of nitrogen (Moyes et al. 2016). Coniferous needles could offer a good habitat for endophytic nitrogen-fixing bacteria, by protecting against adverse environmental conditions and competitors (Yang et al. 2016), and potentially a supply of carbohydrates and energy (Wurzburger 2016). The advantage for conifers would be a potential nitrogen source, either through direct supply from microbe to tree, or through degraded microbial material as a result of a relatively quick microbial turnover (Wurzburger 2016). The possible presence of nitrogen-fixing bacteria inside coniferous needles has been indicated by sequencing data (Carrell and Frank 2014, Haas et al. 2018), and complementary culturing methods have been used to isolate diverse nitrogen-fixing bacteria from a wide range of trees, including some conifers (Doty et al. 2009, Bal et al. 2012, Puri et al. 2018). Usually, their nitrogen-fixing ability is confirmed using either polymerase chain reaction (PCR) amplification to detect the presence of the *nifH* gene, or an acetylene-reduction assay (ARA) to measure their nitrogen-fixation ability (Gupta and Roper 2010, Bal et al. 2012). The most commonly occurring bacterial genera represented in nitrogen-fixing bacteria isolated from coniferous needles are *Sphingomonas*, *Bacillus*, *Rhizobium*, *Pseudomonas*, *Caballeronia* and *Paenibacillus* (Moore et al. 2006, Izumi et al. 2008, Puri et al. 2018). Using ARA, bacterial nitrogen fixation has been measured in needles from limber pine (*Pinus flexilis*) from North American subalpine forest (Moyes et al. 2016), Scots pine (*Pinus sylvestris*) and Norway spruce (*Picea abies*) from the Scandinavian boreal forest (Granhall and Lindberg 1978), and black pine (*Pinus nigra*) and Douglas fir (*Pseudotsuga menziesii*) from Italy (Favilli and Messini 1990). Furthermore, nitrogen fixation has been measured using labelled ^15^N in Douglas fir needles from the temperate forest (Jones 1970). However, the estimated rates of nitrogen fixation between the studies differ significantly, with the amounts of fixed nitrogen ranging from grams to kilograms per hectare and year (Jones 1970, Granhall and Lindberg 1978, Favilli and Messini 1990, Moyes et al. 2016).

In contrast to previously published studies focussing on the presence and activity of nitrogen-fixing bacteria inside needles of native conifer species in the boreal forest, our study is, as far as we know, the first to compare the activity of endophytic nitrogen-fixing bacteria using a nitrogen-addition experiment. The Scots pine was chosen as the study species because, alongside the Norway spruce, it is a dominant tree species in the nitrogen-limited Scandinavian boreal forest (Berlin et al. 2010). The study aimed to confirm the presence of endophytic nitrogen-fixing bacteria inside Scots pine needles, by measuring nitrogen fixation and assessing the effect of nitrogen fertilization on their activity, and isolating and identifying the nitrogen-fixing bacteria. The following hypotheses were addressed: (i) nitrogen-fixing bacteria are present in Scots pine needles; (ii) the bacteria are actively fixing nitrogen and their activity in needles decreases with inorganic nitrogen fertilization; (iii) isolated bacteria, identified by 16S rRNA and whole-genome sequencing, belong to similar genera as found in other conifer species; and (iv) isolated nitrogen-fixing bacteria can fix nitrogen in vitro.

## Materials and methods

### Field site and sample collection

The samples were collected from the Åheden research forest in northern Sweden (N 64° 14′, E 19° 48′). The area in which the field site was located has a mean annual precipitation of 600 mm, a mean annual temperature of 1 °C (Forsmark et al. 2020) and an estimated regional atmospheric nitrogen deposition of less than 2 kg N ha^−1^ year^−1^ (Pihl Karlsson et al. 2012). The Åheden research forest is a naturally regenerated boreal forest dominated by approximately 150-year-old Scots pine (From et al. 2016), with some scattered Norway spruce trees in the sub-canopy. The ground vegetation is predominately lingonberry (*Vaccinium vitis-idaea*), heather (*Calluna vulgaris*), red-stemmed feathermoss (*Pleurozium schreberi*), fork mosses (*Dicranum* spp.), reindeer lichen (*Cladonia rangiferina*) and scrubby cup lichen (*Cladonia arbuscula*) (Gundale et al. 2011). In 2004, five different nitrogen treatments were established at Åheden in 0.1-ha plots; since then the plots have been fertilized yearly with 0, 3, 6, 12 and 50 kg N ha^−1^ year^−1^, respectively, in the form of solid ammonium nitrate granules (NH_4_NO_3_). Each nitrogen treatment has six replicates in a randomized block design (Forsmark et al. 2020).

For this study, the plots fertilized with 0 kg N ha^−1^ year^−1^ (referred to as control plots) and 50 kg N ha^−1^ year^−1^ (referred to as fertilized plots) were selected, as they represented the most contrasting environments at the site. Samples were harvested from two trees from each of the six replicate plots per treatment (*n* = 12 per treatment); tree selection was based on proximity to the centre of the plot and representation of the general appearance, health and size of the trees within the plot. For immunodetection analysis and bacterial isolation, needles from several branches of the same tree were harvested in the summer of 2021, by aseptically collecting 1-year-old (Meredieu 2002) Scots pine needles, which were stored on dry ice for transportation to the laboratory. Several branches from each tree to be used for ARA were aseptically harvested in the summer of 2022 across six sampling days, and stored on ice until analysed in the laboratory. However, at both sampling time points the samples were collected from the same trees and 1-year-old needles were selected as we wanted only healthy green needles.

### Protein extraction and immunodetection of NifH protein

One-year-old Scots pine needles were surface sterilized in a vertical laminar flow hood by submersion in 30% hydrogen peroxide for 2 min with continuous shaking, followed by three washes in sterile deionized water for 20 s. Excess water was evaporated by inverting the samples onto sterile filter paper. The surface-sterilized needles were then stored at −80 °C until the start of the experiment. For total protein isolation, the needles were ground to a fine powder using a pestle and mortar, under constant cooling with liquid nitrogen. Total protein was then extracted by adding 2× protein loading buffer (124 mM Tris–HCl, pH 8.6, 5% sodium dodecyl sulfate (SDS), 4% dithiothreitol and 20% glycerol) to the sample, centrifuging at 4 °C for 5 min at maximum speed, and heating the transferred supernatant at 95 °C for 5 min. Samples were separated by SDS-PAGE (12% Mini-PROTEAN^®^ TGX™ Precast Protein Gels, Bio-Rad, Hercules, CA, USA), with equivalent protein amounts loaded for each sample (measured with a Qubit™ Protein Assay Kit, Invitrogen, Waltham, MA, USA). The separated proteins were transferred onto a nitrocellulose membrane (Amersham Protran, GE Healthcare, Chicago, IL, USA) and stained with Ponceau S as a control for successful transfer. Immunodetection was performed as follows: membranes were blocked for 25 min in 5% (w/v) milk solution dissolved in Tris-buffered saline, 0.1% Tween 20 (TBST) (5% milk powder in 20-mM Tris–HCl, pH 7.4, 180-mM NaCl and 0.1% Tween-20), followed by a 1.5-h incubation at room temperature in a 1:2000 dilution of the primary anti-NifH antibody (Agrisera, Vännäs, Sweden) in TBST containing 2.5% (w/v) milk. After three 15-min wash steps with TBST, the membranes were incubated for 1 h in a dilution of a secondary antibody in TBST containing 2.5% (w/v) milk. The antibody used was horseradish peroxidase-conjugated goat anti-chicken (1:20,000) (Agrisera, Vännäs, Sweden). Again, three 15-min wash steps were performed with TBST. Signal detection was carried out using Pierce™ ECL Western Blotting Substrate (Thermo Fisher Scientific, Waltham, MA, USA) on Azure c600 (Azure Biosystems, Dublin, CA, USA), and quantified using ImageJ (Schneider et al. 2012). To estimate the size of the bands, a PageRuler Prestained Protein Ladder (Thermo Fisher Scientific) was used. To confirm antibody specificity we used positive controls (soybean nodule extract, purified NifH protein with His-tag (Agrisera)) and negative controls (2× protein loading buffer, *Bradyrhizobium japonicum* liquid culture protein extract), which did not yield in a western blot signal.

### Acetylene-reduction assay

The branches harvested for ARA were stored under constant light conditions at room temperature overnight. The next day, for each sample 1-year-old needles were collected from several different branches of the same tree and divided into six subsamples; half of the samples were used to measure acetylene reduction, and half to correct for needle endogenous ethylene production. The needles from each subsample were put into 50 ml glass vials and 8 ml sterile deionized water was added. The ARA was performed as described previously (Richau et al. 2017) with the following modifications: after sealing the glass vials with a rubber septum and replacing 10% of the air with acetylene gas, the samples were incubated at room temperature for 2 h under constant light conditions before 1 ml air was removed with a syringe and analysed for ethylene production on a gas chromatograph (Shimadzu GC-8A, Kyoto, Japan). After the experiment, the needles were dried at 60 °C for 48 h and their dry mass was measured. Because of the high variation in endogenous needle ethylene production observed between individual trees, the ethylene production rates were corrected for sample-specific endogenous ethylene production. Additionally, water samples with injected acetylene were used to correct for any ethylene present due to injected acetylene gas. Specifically, both negative control values were subtracted from the measured acetylene reduction rates from samples, which affected the reported final ethylene production rate.

### Carbon and nitrogen content

Dried 1-year-old Scots pine needles from the ARA were ground to a fine powder in a bead mill (Retsch, Haan, Germany), and the carbon and nitrogen content was measured using an Isotope Ratio Mass spectrometer (DeltaV, Thermo Fisher Scientific) coupled with an Elemental analyser (Flash EA 2000, Thermo Fisher Scientific) (Werner et al. 1999).

### Isolation of nitrogen-fixing bacteria

Half of the 1-year-old Scots pine needles from each sample were surface sterilized by submersion in 30% hydrogen peroxide for 2 min with continuous shaking, and the other half by submersion in 70% ethanol for 3 min with continuous shaking. Two different sterilization methods were used to increase the total number of bacteria isolated. The needles were then washed three times for 20 s with sterile deionized water, and the excess water was removed by inverting the tube onto sterile filter paper. Five needle pairs were imprinted on tryptic soy agar (TSA) plates (15 g l^−1^ casein peptone, 5 g l^−1^ soy peptone, 5 g l^−1^ NaCl and 15 g l^−1^ agar), and the plates were incubated at 28 °C for 10 days to confirm the surface sterility of the samples. The same five needle pairs were ground in phosphate-buffered saline (PBS) buffer (137 mM NaCl, 2.7 mM KCl, 10 mM Na_2_HPO_4_, 1.8 mM KH_2_PO_4_, pH 7.4) using FastDNA Spin Kit tubes and beads and a FastPrep instrument (MP Biomedicals, Irvine, CA, USA). The crude extract was filtered using sterile Miracloth with a pore size of 22–25 μm (Merck Millipore, Burlington, MA, USA), and the filtrate was centrifuged at 5000 r.p.m. for 10 min at 8 °C. The pellet was resuspended in PBS buffer and inoculated on nitrogen-free media (semi-solid NFb (Baldani et al. 2014), combined carbon media (CCM) without yeast extract (Baldani et al. 2014) and LGI-P (Reis et al. 2015). The plates (CCM and LGI-P) and tubes (NFb) were incubated at 28 °C for 10 days. Individual colonies were observed and re-cultured on TSA plates.

### 16S rRNA and whole-genome bacterial sequencing

For 16S rRNA Sanger sequencing of isolated bacteria, the bacteria were grown on TSA plates overnight. Bacterial material was then transferred into an extraction buffer (0.05 M NaOH, 0.25% SDS), which was heated at 97 °C for 15 min before the sample was centrifuged for 4 min at 10,000 r.p.m. The collected supernatant was diluted with Tris-EDTA (TE) buffer (10 mM Tris, 1 mM EDTA, pH 8.0) and the extract was used in a PCR with the 16S ribosomal ribonucleic acid (rRNA) primers 27F/1492R (Heuer et al. 1997). The PCR was carried out using a DreamTaq Hot Start PCR Master Mix (Thermo Fisher Scientific) according to the manufacturer’s instructions, with 1 μl of bacterial extract, 0.5 μM forward and reverse primers, and an annealing temperature of 55 °C. Based on agarose gel separation, all the isolated bacteria had a band of around 1500 bp, so the PCR products were cleaned with ExoSAP-IT PCR Product Cleanup (Applied Biosystems, Waltham, MA, USA) before tube Sanger sequencing (Eurofins, Luxembourg City, Luxembourg). The obtained sequences were analysed using Basic Local Alignment Search Tool (BLAST) (Altschul et al. 1990) and compared with sequences already in the National Center for Biotechnology Information (NCBI) database. A phylogenetic tree showing the similarities between the strains was produced using Phylogeny.fr (Dereeper et al. 2008) according to Gratz et al. (2021), using MUSCLE alignment, Gblocks curation, PhyML phylogeny and TreeDyn tree rendering, but with likelihood-ratio test (minimum of SH-like and Chi2-based) instead of bootstrapping.

For whole genome sequencing, liquid Luria broth (LB) media (10 g l^−1^ tryptone, 5 g l^−1^ yeast extract and 10 g l^−1^ NaCl) was inoculated with isolated bacteria overnight before being centrifuged for 5 min at 10,000 r.p.m. The pellet was resuspended in a smaller amount of liquid LB media and the deoxyribonucleic acid (DNA) was isolated using a DNeasy PowerSoil Kit (Qiagen, Venlo, The Netherlands). The amount and quality of the DNA were assessed using Nanodrop before being sent for sequencing and bioinformatic analysis (CD Genomics, New York City, NY, USA through Genohub, Austin, TX, USA). For sequencing, an Illumina NovaSeq6000 (Illumina, San Diego, CA, USA) was used with pair-end 2× 150 base pair sequencing and at least 3 million reads per sample. For the bioinformatic analysis, the two pairs of reads were merged, 1000 sequences were randomly selected, and BLAST (Altschul et al. 1990) was used to compare the obtained sequences with sequences already in the NCBI database (NCBI, Bethesda, MD, USA). The identity was determined based on the sequence hit count. Additionally, Read Assembly and Annotation Pipeline Tool (RAPT) (NCBI, Bethesda, MD, USA) was used for de novo assembly of the bacterial genomes using SKESA and annotation of the genome using Prokaryotic Genome Annotation Pipeline to check for the presence of *nif* genes within the whole genome sequence.

The bacterial 16S rNRA sequences were deposited in GenBank (NCBI), and the unassembled Illumina whole genome sequences were deposited in the Sequence Read Archive (NCBI) (Sayers et al. 2022) (Table S1 available as Supplementary data at *Tree Physiology* Online). Bacterial cultures were deposited in the NCCB collection (Westerdijk Fungal Biodiversity Institute, Utrecht, The Netherlands) (Table S1 available as Supplementary data at *Tree Physiology* Online).

### In vitro nitrogen fixation of isolated bacteria

The nitrogen-fixation ability of isolated bacteria was measured by ARA. Bacteria were grown in either liquid CCM without yeast extract (bacteria #1, 23, 24, 25, 27, 39) or liquid LGI-P media (bacteria #2, 3, 14, 26, 28, 38-1, 38-2) for 24 h at 28 °C, and then their OD_600_ measured. The bacteria were transferred to glass vials sealed with a rubber septum; 10% of the air was replaced by acetylene and, after a 2-h incubation at 30 °C with constant shaking, the ethylene production was measured using a gas chromatograph (Shimadzu GC-8A, Kyoto, Japan). The bacterial OD_600_ was measured again after the experiment and this value was used to normalize the ethylene production. Calculated ethylene production rates were corrected for spontaneous acetylene reduction and any endogenous ethylene production by either the media or the bacteria.

### Statistics

All data were analysed using SPSS Statistics 27 (IBM, Armonk, NY, USA). For data from the immunodetection of NifH protein, ARA on needles, in vitro ARA on bacterial cultures, and carbon and nitrogen content, the assumptions of normal distribution and equal variance were checked. For all datasets, both assumptions were met, as well as the assumption of independence. The data from the immunodetection of NifH protein, and carbon and nitrogen content, were then analysed using a two-sample independent *t*-test. In vitro ARA measurements on isolated bacteria were analysed using a one-way analysis of variance (ANOVA) followed by a Tukey honestly significant difference (HSD) test. For the ARA on needles, a two-way ANOVA was used, using sampling day and nitrogen treatment as variables. A linear regression model was used to analyse the relationship between ARA in 1-year-old Scots pine needles and the C/N ratio. The regression coefficient was tested to see whether there was a statistically significant relationship between the two variables.

## Results

**Figure 1:**
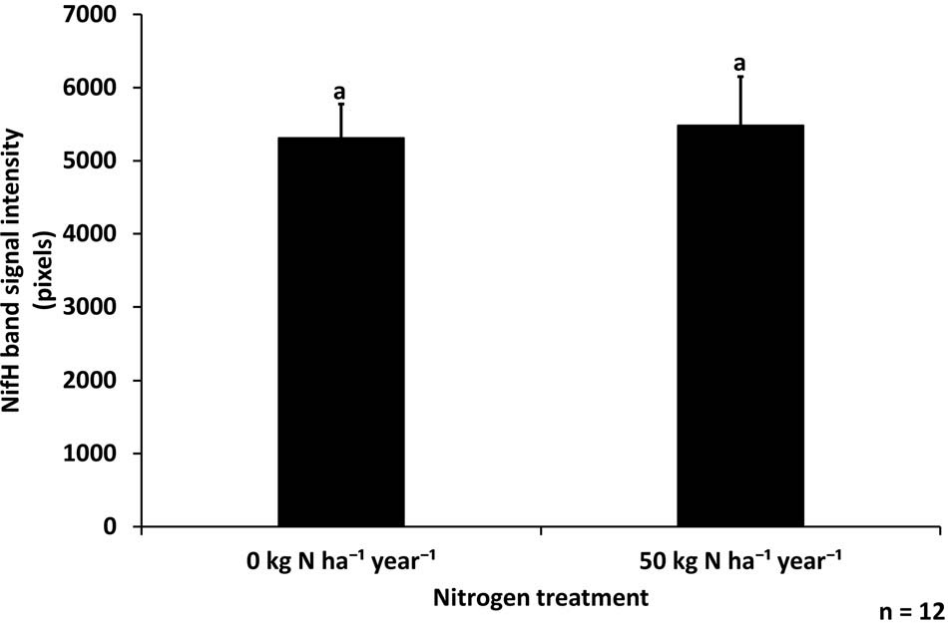
NifH band signal intensity for immunodetection with anti-NifH antibody (mean + SE), indicating the presence of nitrogenase enzyme in 1-year-old Scots pine needles from trees grown in control and nitrogen-fertilized plots (0 and 50 kg N ha^−1^ year^−1^, respectively) (*n* = 12 per treatment, two-sample *t*-test *P* = 0.84, bars with different letters are significantly different).

The presence of nitrogen-fixing bacteria inside surface-sterilized needles was analysed using immunoblotting of the NifH protein, which is one of the nitrogenase enzyme’s subunits. Our analyses indicated that nitrogen-fixing bacteria were present in the needles from both control and nitrogen-fertilized plots (Table S2 available as Supplementary data at *Tree Physiology* Online, Figure 1), and the signal intensity of the NifH band was similar between the two treatments (two-sample *t*-test, *P* = 0.84), with values of 5309 and 5481, respectively. This indicated a similar amount of nitrogenase protein in both treatments (Figure 1).

**Figure 2:**
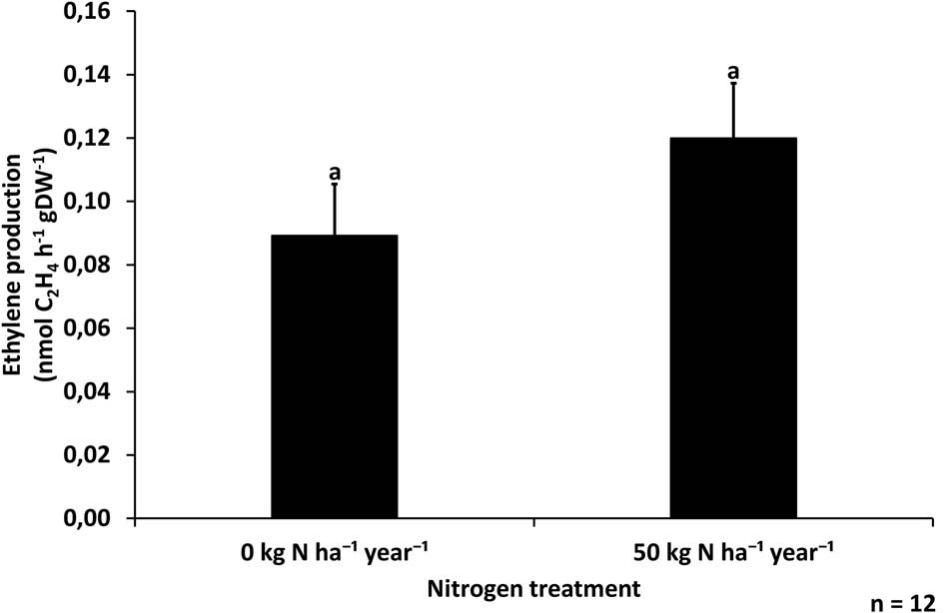
Ethylene production rate (mean + SE) per hour and gram dry mass of 1-year-old Scots pine needles, indicating nitrogenase enzyme activity in the needles of trees gown in control and nitrogen-fertilized plots (0 and 50 kg N ha^−1^ year^−1^, respectively) (*n* = 12 per treatment, two-sample *t*-test *P* = 0.11, bars with different letters are significantly different).

As the presence of nitrogenase protein does not mean active nitrogen fixation, ARA was used to measure the nitrogenase activity indirectly. As well as reducing dinitrogen to ammonia, nitrogenase enzymes can also reduce acetylene to ethylene, which can be detected with this method. We measured the nitrogenase activity in needles from both the control plots and inorganic nitrogen-fertilized plots (Table S3 available as Supplementary data at *Tree Physiology* Online). The average nitrogenase activity was slightly higher in needles from fertilized plots than in control plots (Figure 2), but the difference was not significant (two-way ANOVA, *P* = 0.11). The fixation rate for needles from control plots was 0.09 nmol ethylene h^−1^ and g^−1^ dry needles, and the rate for fertilized plots was 0.12 nmol ethylene h^−1^ and g^−1^ dry needles. There was, however, a significant effect of sampling day, with a difference in nitrogen fixation rates between different sampling dates (two-way ANOVA, *P* = 0.02), highlighting the importance of including a sufficient number of negative controls in the measurement protocol for each sampling date.

To determine the possible relevance of the nitrogen-fixation activity for the forest, the nitrogen-fixation rates were extrapolated to provide an estimate for the forest stand. We assumed equal nitrogen-fixation rates across the whole canopy regardless of needle position, an equal rate of nitrogen fixation during the whole growth period, as measured in our study, a 150-day growth period (Goude et al. 2019), a constant 12-h period of daylight (Moyes et al. 2016), and a conversion factor between ethylene production and nitrogen fixation of 3:1 (Hardy et al. 1968). Using an average leaf area index for Scots pine forest across the Swedish boreal forest (Appiah Mensah et al. 2020), the calculated nitrogen-fixation rate for control plots was approximately 11 g N ha^−1^ year^−1^, and for fertilized plots, it was 15 g N ha^−1^ year^−1^.

The carbon and nitrogen content of the needles was investigated to see whether they could affect the nitrogen fixation rates. The average carbon content for needles from the control plots was 51.0 g C g^−1^ dry mass and from fertilized plots 51.5 g C g^−1^ dry mass, which was not statistically significant (two-sample *t*-test, *P* = 0.07). The nitrogen content did differ between the two treatments, however, with average nitrogen content in needles from control plots of 0.93 g N g^−1^ dry mass, and from fertilized plots of 1.37 g N g^−1^ dry mass. This difference was statistically significant (two-sample *t*-test, *P* < 0.01). There was no significant linear regression relationship between the measured C/N ratios and ethylene production rates (ethylene production rates = −0.0018 × C/N ratio + 0.19, *R*^2^ = 0.097, *P* = 0.14) of the needles.

**Figure 3:**
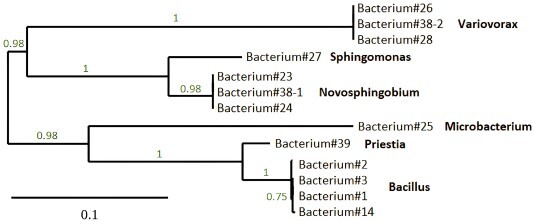
A phylogenetic tree (produced using Phylogeny.fr) of the 13 potentially nitrogen-fixing bacteria, based on 16S rRNA sequencing. The numbers represent the likelihood-ratio test values of the branching points.

To identify bacteria possibly responsible for the nitrogenase protein content and activity, potential nitrogen-fixing bacteria were isolated from surface-sterilized needles from trees grown in either control or nitrogen-fertilized plots. Three different nitrogen-free media (CCM, LGI-P and NFb) were used, and 13 distinct bacterial colonies were successfully isolated. The bacteria were identified using 16S rRNA Sanger sequencing and Illumina whole-genome sequencing. The 16S rRNA sequences revealed that the isolated bacteria belonged to several different genera: *Bacillus*, *Microbacterium*, *Variovorax*, *Priestia*, *Novosphingobium* and *Sphingomonas* (Figure 3). More specifically, whole-genome sequencing identified the bacteria isolated from control plots as three *Bacillus paralicheniformis*, two unclassified *Novosphingobium*, two *Variovorax paradoxus*, one *Microbacterium* sp. and one *Sphingomonas* sp. (Table 1). Of the bacteria isolated from nitrogen-fertilized plots, one was identified as *Priestia megaterium*, one as *B. paralicheniformis*, one as *V. paradoxus* and one as *Novosphingobium pokkalii* (Table 1). The presence of *nif* genes was looked at using the assembled and annotated genome and we could detect the presence of sequence for NifU protein in bacteria 1, 2, 3, 14, 23, 24, 27, 38-1 and 39.

**Table 1:** Potential nitrogen-fixing bacteria isolated from 1-year-old Scots pine needles. The trees were grown in control plots (0 kg N ha^−1^ year^−1^) or long-term nitrogen-fertilized plots (50 kg N ha^−1^ year^−1^), and the bacteria were isolated on different nitrogen-free media (CCM, LGI-P or NFb). The genus of each isolated bacterium was determined by whole-genome sequencing.

Bacteria	Nitrogen treatment	Plate	Species
1	0 kg N ha^−1^ year^−1^	CCM	*Bacillus paralicheniformis*
23	0 kg N ha^−1^ year^−1^	CCM	Unclassified *Novosphingobium*
24	0 kg N ha^−1^ year^−1^	CCM	Unclassified *Novosphingobium*
25	0 kg N ha^−1^ year^−1^	CCM	*Microbacterium* sp.
27	0 kg N ha^−1^ year^−1^	CCM	*Sphingomonas* sp.
2	0 kg N ha^−1^ year^−1^	LGI-P	*Bacillus paralicheniformis*
3	0 kg N ha^−1^ year^−1^	LGI-P	*Bacillus paralicheniformis*
26	0 kg N ha^−1^ year^−1^	LGI-P	*Variovorax paradoxus*
28	0 kg N ha^−1^ year^−1^	NFb	*Variovorax paradoxus*
39	50 kg N ha^−1^ year^−1^	CCM	*Priestia megaterium*
14	50 kg N ha^−1^ year^−1^	LGI-P	*Bacillus paralicheniformis*
38-1	50 kg N ha^−1^ year^−1^	NFb	*Novosphingobium pokkalii*
38-2	50 kg N ha^−1^ year^−1^	NFb	*Variovorax paradoxus*

**Figure 4:**
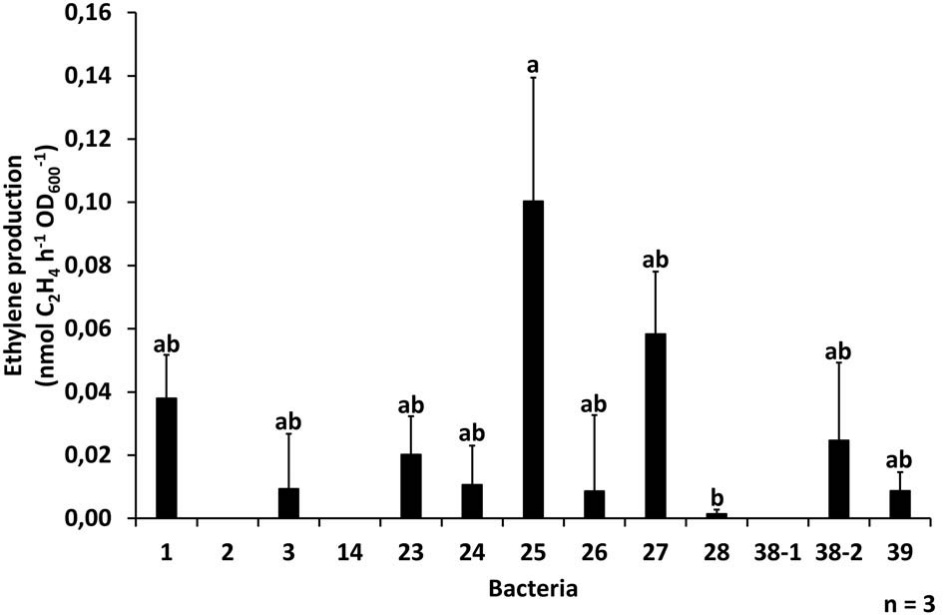
The nitrogenase activity, measured indirectly through ethylene production, of liquid cultures of 13 isolated bacteria (mean + SE). Bars with different letters are significantly different (one-way ANOVA followed by Tukey HSD test, *P* < 0.05).

To confirm the ability of the 13 isolated bacteria to fix nitrogen, ethylene production was measured during ARA on liquid bacterial cultures. Ten of the 13 isolated bacterial colonies were able to fix nitrogen under specific conditions, producing ethylene to various degrees (Figure 4). Bacterium 25 (*Microbacterium* sp.) had the highest nitrogenase activity, whereas bacteria 2 (*B. paralicheniformis*), 14 (*B. paralicheniformis*) and 38-1 (*N. pokkalii*) were not capable of nitrogen fixation under the test conditions.

## Discussion

It has been suggested that endophytic nitrogen fixation in conifer needles could be prevalent across temperate and boreal forests (Moyes et al. 2016), and even a small amount of nitrogen fixed by these bacteria could be ecologically important in nitrogen-limited environments (Wurzburger 2016). The main aim of our study was, therefore, to determine the presence, and measure of the activity, of endophytic nitrogen-fixing bacteria inside 1-year-old needles from Scots pine trees growing in the Scandinavian boreal forest.

By the first hypothesis, bacterial nitrogenase protein was detected in all of the samples analysed, indicating the widespread presence of nitrogen-fixing bacteria in Scots pine needles in this experimental forest area. The needles were surface sterilized before analysis, so the bacterial presence was probably endophytic. There seemed to be a similar abundance of the bacteria in the needles, as the amount of nitrogenase protein did not differ significantly between needles from control and nitrogen-fertilized plots.

The activity of nitrogen-fixing bacteria was measured using ARA. Our study corroborates results reported elsewhere that coniferous needle ethylene production can vary substantially as a result of needle age, position, season and sterilization protocol (Telewski 1992, Ievinsh and Tillberg 1995, Ievinsh and Ozola 1998, Klintborg et al. 2002). Because endogenous ethylene production was highly variable between the trees, we ensured that sample-specific production rates were measured and subtracted from the ARA ethylene production rates. The nitrogen-fixation rates were around 10 times higher than previously reported for the phyllosphere of Scots pine (Granhall and Lindberg 1978), which could be explained by different environmental or experimental factors between the two studies. It was, however, slightly lower, although in the same range, as rates reported for limber pine needles (Moyes et al. 2016), and much lower than rates reported for black pine and Douglas fir needles (Jones 1970, Favilli and Messini 1990). The nonsignificant difference in nitrogen-fixation rates between needles from trees on control and fertilized plots contradicted our second hypothesis that inorganic nitrogen fertilization would decrease the activity of nitrogen-fixing bacteria. This result is to some extent unexpected, as many studies have reported decreased nitrogen-fixation activity in biotopes enriched with inorganic nitrogen. For instance, from the same site as studied here, the nitrogen fixation of cyanobacteria associated with ground-dwelling moss surfaces was approximately eight times lower on fertilized than on control plots (Gundale et al. 2011). The same trend of decreased nitrogen fixation in response to nitrogen fertilization has also been observed in soil, forest floor and tree canopy leaves of disturbed subtropical forests in China, where the decrease between the two treatments was between 20 and 38% (Zheng et al. 2017). Additionally, it has been reported that nitrogen fixation in root nodules of individual legume trees (*Inga* sp.) from tropical forests grown in soils with a higher nitrogen content is almost nonexistent compared with those grown in soils with a lower nitrogen content (Barron et al. 2011). However, there are also studies showing no clear effect of nitrogen availability or fertilization on nitrogen fixation. For example, no correlation was found between endophytic nitrogen fixation and plant-available soil nitrogen for limber pine needles (Moyes et al. 2016). Also, in a rehabilitated subtropical forest, there was no significant effect of nitrogen fertilization on nitrogen fixation in soil and tree canopy leaves (Zheng et al. 2017). Furthermore, using a mathematical model it was suggested that in nitrogen-poor boreal forests obligate nitrogen-fixing bacteria could be prevalent due to the high cost of being facultative (Menge et al. 2009), which could explain the observed no difference in nitrogen fixation rates between control and fertilized plots.

The mechanism behind the influence of external plant nitrogen sources on nitrogen-fixation rates is not fully understood. In the case of needle endophytic nitrogen fixation for trees in a strictly nitrogen-limited environment, one speculation is that fertilization can enhance photosynthesis by increasing the supply of energy available for nitrogen fixation. Alternatively, the opposite could also be argued, as a higher C/N ratio arising from fertilization may mean that the externally supplied nitrogen renders nitrogen fixation redundant. However, in this study, whereas the C/N ratio of the needles significantly decreased with nitrogen fertilization, there was no significant relationship between the needles’ C/N ratio and the nitrogen fixation rates of the needles (*P* = 0.14). A similar result has been found for logging residues of Scots pine, Norway spruce and silver birch (*Betula pendula*), where neither branches nor foliage showed a significant correlation between nitrogen fixation and C/N ratio (Tormanen and Smolander 2022).

To understand better the potential importance of needle nitrogen fixation in a nitrogen-limited forest, we extrapolated our measured nitrogen-fixation rates to a forest scale, calculating it to be less than 20 g N ha^−1^ year^−1^. However, this rate needs to be interpreted with caution, as several assumptions were made, including constant nitrogen fixation during the growth season, which could be incorrect as seasonal variation in conifers has been noticed elsewhere (Favilli and Messini 1990). Our study also indicates that there is significant day-to-day variation in nitrogen-fixation rates, which was not accounted for in our extrapolation. With these caveats in mind, the estimated nitrogen contributed by nitrogen fixation in needles seems very low compared with the estimated nitrogen use of approximately 50 kg N ha^−1^ year^−1^ for Scots pine growth in the boreal forest (Korhonen et al. 2013). The contribution of needle nitrogen fixation to tree growth in this forest may, therefore, be rather insignificant in the short term. However, taking into account the approximately 8500-year continuous boreal forest cover in the region (Barnekow et al. 2008), and assuming historically constant nitrogen fixation in needles, the nitrogen fixed by bacteria inside Scots pine needles could be an important nitrogen source in the longer term, contributing to the build-up of the soil nitrogen stock over the long term (Finér et al. 2003, Merilä et al. 2014).

Largely consistent with our third hypothesis, the endophytic bacteria isolated from 1-year-old Scots pine needles belonged to similar genera as found in other tree species (Cankar et al. 2005, Moore et al. 2006, Izumi et al. 2008, Puri et al. 2018). The 13 isolated bacteria colonies were identified as belonging to the genera *Bacillus*, *Variovorax*, *Novosphingobium*, *Sphingomonas*, *Microbacterium* and *Priestia*. The genera *Penibacillus*, *Rhizobium* and *Pseudomonas* were not present, even though they are commonly found in tree species (Cankar et al. 2005, Moore et al. 2006, Izumi et al. 2008, Puri et al. 2018). Looking more closely at each genus, *Bacillus* bacteria have been detected previously in various coniferous and deciduous trees (Izumi et al. 2008, Puri et al. 2018), and have been shown to fix nitrogen and promote plant growth and yield (Çakmakçı et al. 2001, Ding et al. 2005, Yousuf et al. 2017). The Gram-positive, rod-shaped (Dunlap et al. 2015) strain of *B. paralicheniformis* has been reported as a nitrogen-fixing bacterium based on a whole-genome study (Annapurna et al. 2018). Another of the isolated bacteria belonged to the genus *Microbacterium*, which includes Gram-positive bacteria that have been shown to promote plant growth, chlorophyll content and fruit yield in a few diverse but agriculturally important plant species (Karlidag et al. 2007, Schwachtje et al. 2012, Mutai et al. 2017, Bal and Adhya 2021). This genus includes strains with the *nifH* gene and the capacity to fix nitrogen (Ruppel 1989, Zakhia et al. 2006, Lin et al. 2012), and some *Microbacterium* strains have been isolated from maple and elm trees (Shen and Fulthorpe 2015). Bacteria from *Variovorax* have been isolated from poplar trees (Moore et al. 2006), and this genus of Gram-positive bacteria includes strains capable of nitrogen fixation (Solanki et al. 2016) and promoting plant growth (Maimaiti et al. 2007). Specifically, *V. paradoxus* has been reported as a hydrogen-oxidizing plant growth-promoting bacterium (Maimaiti et al. 2007, Han et al. 2011). The Gram-positive *P. megaterium* (previously classified as *Bacillus megaterium*) has also been reported as a nitrogen-fixing bacterium (Ding et al. 2005, Yousuf et al. 2017) and shown to promote plant growth (Nascimento et al. 2020, Wang et al. 2021). *Sphingomonas* strains have been detected in willow and elm trees (Moore et al. 2006, Doty et al. 2009, Shen and Fulthorpe 2015), and the ability to fix nitrogen has been identified in a few *Sphingomonas* bacteria (Castanheira et al. 2014, Yang et al. 2014, Lowman et al. 2015). *Novosphingobium* has also been reported as a genus that includes plant growth-promoting nitrogen-fixing bacteria (Islam et al. 2009, Rangjaroen et al. 2017). *Novosphingobium pokkalii* has been described as a rhizosphere-associated bacterium with plant growth-promoting properties (Krishnan et al. 2017).

Acetylene-reduction assay (ARA) was used to check in vitro whether the isolated bacteria were endophytic diazotrophic bacteria capable of nitrogen fixation. In support of our fourth hypothesis, 10 out of the 13 bacteria colonies displayed ethylene production, with *Microbacterium* sp. being the most efficient. The three colonies that did not fix nitrogen in the ARA were identified as two species, *B. paralicheniformis* and *N. pokkalii*. It could be that these three bacterial strains did not show nitrogen fixation because of nonoptimal test conditions (Doty et al. 2009), or because their fixation rate was under the detection limit of the ARA. Not all bacteria capable of growing on nitrogen-free media demonstrate nitrogen fixation during an ARA (Doty et al. 2009, Padda et al. 2018, Puri et al. 2018). Using assembled and annotated genomes of the bacteria we could detect the sequence for the NifU protein in most of the bacteria, however, we could not detect any other *nif* genes in the sequences. This could be due to short reads limitation (as we only had 150 bp sequencing length), genome misassemblies or genome and annotation incompleteness (Chen et al. 2013, Barbitoff et al. 2020, Lobb et al. 2020).

The fact that most of the isolated endophytic bacteria were capable of nitrogen fixation makes them good candidates for plant growth-promoting bacteria. Nitrogen-fixing bacteria isolated from conifers have been used as plant growth-promoting bacteria in seedlings: the inoculated seedlings were taller and had greater biomass compared with control seedlings grown under both nitrogen-limited conditions (Puri et al. 2020) and in fertilized soil (Chen et al. 2021). However, to analyse their potential as plant growth-promoting bacteria our isolated strains would need to be tested for additional plant growth-promoting properties, such as indole-3-acetic acid production, siderophore production and 1-aminocyclopropane-1-carboxylate deaminase.

## Conclusions

Our study has demonstrated that nitrogen-fixing bacteria are present and active in 1-year-old Scots pine needles; their endophytic presence was confirmed by nitrogenase protein immunodetection, and nitrogen fixation was measured using ARA. Strains of the nitrogen-fixing bacteria were isolated from sterile needles by culturing and identified using whole-genome sequencing, and their nitrogen-fixation ability was confirmed by in vitro ARA. Immunodetection of the NifH protein showed no difference between needles from control plots and fertilized plots, and the ARA showed similar fixation rates in needles from both treatments. To scale up estimates of the nitrogen-fixation rates and their impact on the boreal forest more accurately, it is important that the seasonality of nitrogen fixation is assessed, and the effect of variation in light intensity on the nitrogen-fixation capacity of endophytic bacteria inside coniferous needles determined. Even though the amount of nitrogen fixed by these bacteria might not be significant for the trees currently growing in the Scandinavian boreal forest, it could be significant in the longer term. Nitrogen fixation by bacteria within conifer needles may have provided an important source of nitrogen for the forest ecosystem’s structure and function during the millennia that the boreal forest has dominated this landscape.

## Supplementary Material

Supplementary_tables_1-3_tpad048

Supplementary_data-clean_copy_tpad048

## Data Availability

Sequencing data and bacterial strains are made available through GenBank, Sequence Read Archive and NCCB collection.
