# Supplementary material for: Presence and activity of nitrogen-fixing bacteria in Scots pine needles in a boreal forest: a nitrogen-addition experiment
Source: Tree Physiol. 2023 Apr 18;43(8):1354–64. doi: 10.1093/treephys/tpad048 (PMC10423461; doi:10.1093/treephys/tpad048)
Supplement: Supplementary_data-clean_copy_tpad048 [file supplementary_data-clean_copy_tpad048.docx]

**Supplementary data**

**Supplementary Table 1:** Accession numbers for the deposits made to GenBank (16S rRNA sequences), the Sequence Read Archive (unassembled Illumina whole genome sequences) and the NCCB collection (bacterial cultures) derived from the 13 colonies isolated during the study.


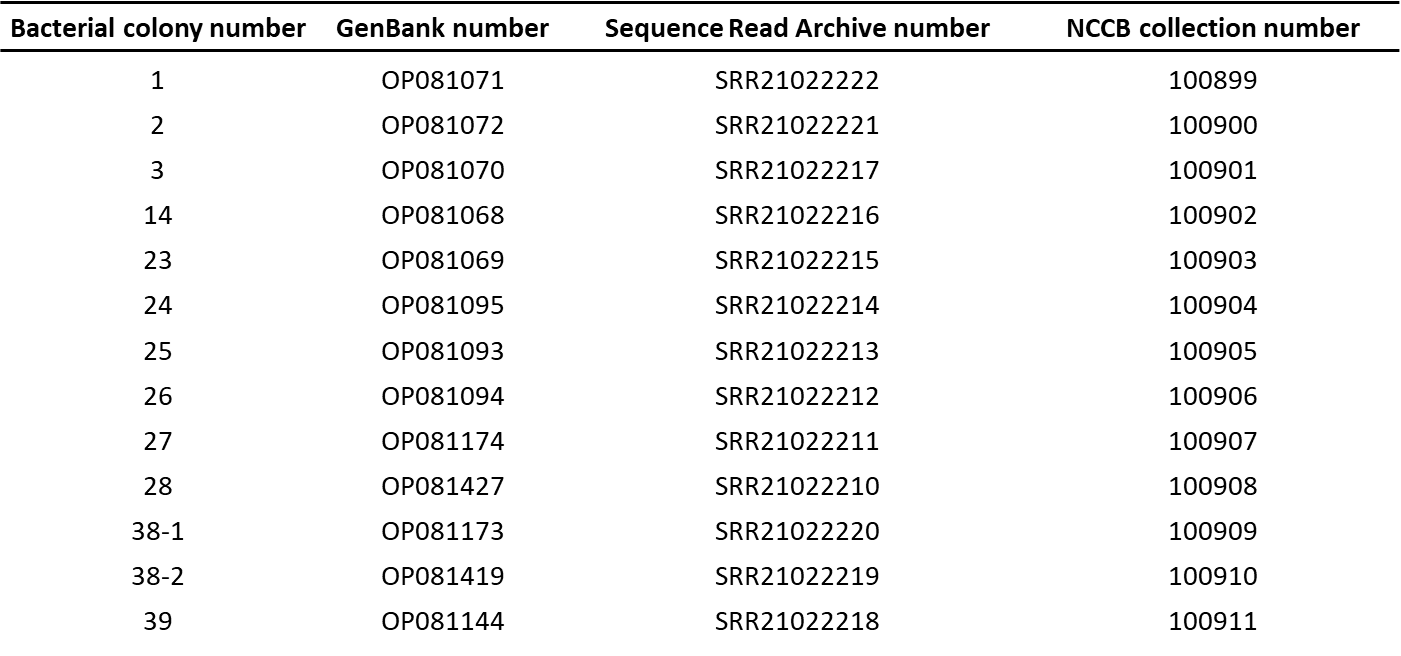


**Supplementary Table 2**: The presence of nitrogenase enzyme, as indicated by the signal intensity of the NifH band based on anti-NifH antibody immunodetection, in one-year-old needles from Scots pine trees grown in control (0 kg N ha^–1^ year^–1^) and long-term inorganic nitrogen-fertilized (50 kg N ha^–1^ year^–1^) plots. The samples were taken from two trees in each of six replicate plots per treatment.


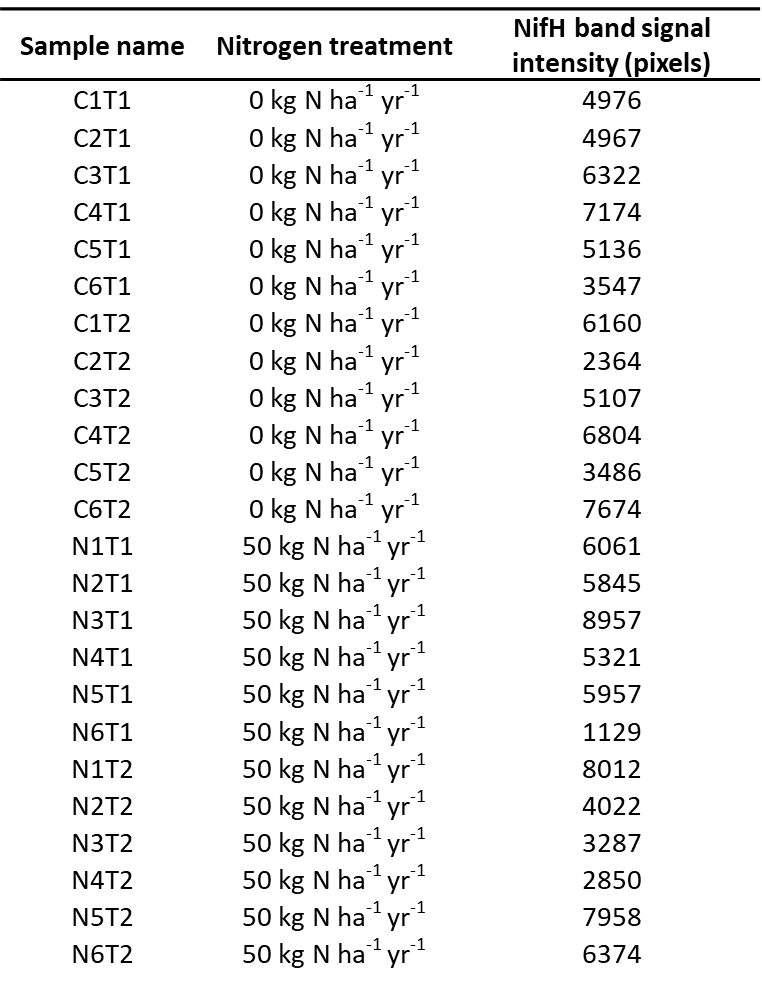


**Supplementary Table 3**: Measured sample ethylene production rates, sample-specific negative control ethylene production rates and final calculated ethylene production rates with corresponding standard error values, indicating nitrogenase enzyme activity, per gram dry mass of one-year-old needles from Scots pine trees grown in control (0 kg N ha^–1^ year^–1^) and long-term inorganic nitrogen-fertilized (50 kg N ha^–1^ year^–1^) plots. The samples were taken from two trees in each of six replicate plots per treatment and all results are averages from three biological replicates.

| **Sample name** | **Nitrogen treatment** | **Sample ethylene production (nmol C_2_H_4_ h^-1^ g^-1^)** | **Negative control ethylene production (nmol C_2_H_4_ h^-1^ g^-1^)** | **Final calculated ethylene production (nmol C_2_H_4_ h^-1^ g^-1^)** | **Standard error for final calculated ethylene production (nmol C_2_H_4_ h^-1^ g^-1^)** |
| --- | --- | --- | --- | --- | --- |
| C1T1 | 0 kg N ha^-1^ yr^-1^ | 0.231 | 0.193 | 0.037 | 0.056 |
| C2T1 | 0 kg N ha^-1^ yr^-1^ | 0.189 | 0.111 | 0.078 | 0.023 |
| C3T1 | 0 kg N ha^-1^ yr^-1^ | 0.127 | 0.093 | 0.034 | 0.004 |
| C4T1 | 0 kg N ha^-1^ yr^-1^ | 0.427 | 0.461 | -0.034 | 0.025 |
| C5T1 | 0 kg N ha^-1^ yr^-1^ | 0.193 | 0.077 | 0.116 | 0.007 |
| C6T1 | 0 kg N ha^-1^ yr^-1^ | 0.302 | 0.212 | 0.090 | 0.007 |
| C1T2 | 0 kg N ha^-1^ yr^-1^ | 0.165 | 0.083 | 0.082 | 0.019 |
| C2T2 | 0 kg N ha^-1^ yr^-1^ | 0.383 | 0.248 | 0.135 | 0.018 |
| C3T2 | 0 kg N ha^-1^ yr^-1^ | 0.317 | 0.164 | 0.153 | 0.027 |
| C4T2 | 0 kg N ha^-1^ yr^-1^ | 0.346 | 0.200 | 0.146 | 0.046 |
| C5T2 | 0 kg N ha^-1^ yr^-1^ | 0.456 | 0.372 | 0.084 | 0.030 |
| C6T2 | 0 kg N ha^-1^ yr^-1^ | 0.413 | 0.263 | 0.150 | 0.024 |
| N1T1 | 50 kg N ha^-1^ yr^-1^ | 0.232 | 0.178 | 0.053 | 0.029 |
| N2T1 | 50 kg N ha^-1^ yr^-1^ | 0.442 | 0.355 | 0.086 | 0.014 |
| N3T1 | 50 kg N ha^-1^ yr^-1^ | 0.173 | 0.102 | 0.071 | 0.004 |
| N4T1 | 50 kg N ha^-1^ yr^-1^ | 0.276 | 0.189 | 0.087 | 0.008 |
| N5T1 | 50 kg N ha^-1^ yr^-1^ | 1.420 | 1.133 | 0.287 | 0.130 |
| N6T1 | 50 kg N ha^-1^ yr^-1^ | 0.259 | 0.140 | 0.120 | 0.019 |
| N1T2 | 50 kg N ha^-1^ yr^-1^ | 0.412 | 0.277 | 0.136 | 0.043 |
| N2T2 | 50 kg N ha^-1^ yr^-1^ | 0.185 | 0.098 | 0.087 | 0.020 |
| N3T2 | 50 kg N ha^-1^ yr^-1^ | 0.202 | 0.102 | 0.100 | 0.006 |
| N4T2 | 50 kg N ha^-1^ yr^-1^ | 0.333 | 0.193 | 0.140 | 0.019 |
| N5T2 | 50 kg N ha^-1^ yr^-1^ | 0.298 | 0.170 | 0.128 | 0.047 |
| N6T2 | 50 kg N ha^-1^ yr^-1^ | 0.499 | 0.354 | 0.145 | 0.019 |
